# A study protocol for a baseline community-based cross-sectional study to investigate malaria and dengue transmission dynamics in a development area of new capital city, Indonesia

**DOI:** 10.12688/wellcomeopenres.25303.1

**Published:** 2026-02-03

**Authors:** Alfa Pradana, Margareta Oktaviani, Hellen Prameswari, Dedy Supriyanto, Ponco Waluyo, Ermi Ndoen, Dian Rosadi, Inke Lubis, Suwarti Suwarti, Decy Subekti, Tina Kusumaningrum, Miles Carroll, Bimandra Djaafara, Ruklanthi de Alwis, Swapnil Mishra, Chris Drakeley, Karin Leder, Alex Lechner, Kimberly Fornace, Iqbal Elyazar, Henry Surendra

**Affiliations:** 1 Infectious and Tropical Diseases Epidemiology, Public Health Program, Monash University Indonesia, Tangerang, Indonesia; 2 Oxford University Clinical Research Unit Indonesia, Faculty of Medicine, Universitas Indonesia, Jakarta, Indonesia; 3 Ministry of Health of Indonesia, Jakarta, Indonesia; 4 District Health Office of Penajam Paser Utara, East Kalimatan, Indonesia; 5 UNICEF, Jakarta, Indonesia; 6 Department of Epidemiology, Public Health Study Program, Faculty of Medicine, Lambung Mangkurat University, Banjarbaru, Indonesia; 7 Faculty of Medicine, Universitas Sumatera Utara, Medan, Sumatera Utara, Indonesia; 8 Pandemic Sciences Institute & Centre for Human Genetics, University of Oxford, Oxford, UK; 9 Saw Swee Hock School of Public Health, National University of Singapore, Singapore, Singapore; 10 Emerging Infectious Disease, Duke-NUS Medical School, Singapore, Singapore; 11 Department of Infection Biology, London School of Hygiene & Tropical Medicine, London, UK; 12 Division of Planetary Health, School of Public Health and Preventive Medicine, Monash University, Melbourne, Australia; 13 Health and Climate Initiative, Faculty of Medicine Nursing and Health Sciences, Monash University, Melbourne, Australia

**Keywords:** malaria, dengue, serology, seroprevalence, cross-sectional study, Ibu Kota Nusantara, surveillance

## Abstract

**Background:**

The relocation of Indonesia's capital to Ibu Kota Nusantara (IKN) in East Kalimantan, a malaria and dengue hotspot, presents new risks of infectious disease transmission due to land-use changes and population movements. Current knowledge on the impact of these changes on vector-borne diseases, especially *Plasmodium knowlesi* malaria and other arboviruses, is limited. Serological surveillance offers a robust method for assessing population exposure.

**Method:**

A community-based cross-sectional study will be conducted in IKN and its surrounding area, in East Kalimantan. Approximately 2,000 individuals aged >1 year will be enrolled. Finger-prick blood samples will be collected for serological analysis (multiplex bead-based assays for malaria species, and dengue virus serotypes) and malaria RDTs. Demographic, clinical, environmental, and geolocation data will also be collected. Statistical and geostatistical models will be used to assess seroprevalence, spatial patterns, and risk factors of exposure to malaria and dengue.

## Plain Language Summary

Our study aims to understand the historical transmission risk of malaria and dengue among communities living in and around the new capital city. We will recruit around 2,000 people, aged ≥1 year old from randomly selected households in a development area of new capital city, Indonesia.

From each consenting participant, we will take a finger-prick blood sample. This sample will be used for two purposes: first, a malaria diagnostic test (RDT), so we can refer to anyone who is ill for immediate treatment. Second, we will use laboratory tests to look for antibodies in the blood. Antibodies are a sign that a person’s immune system has been exposed to infections (or vaccinations) in the past. In our study, we aim to estimate the proportion of the population who have been exposed to malaria or dengue infections in the past, even if they never felt ill.

By integrating health data with satellite-derived environmental data, we will identify hotspots where the risk of infection is highest and understand the factors that drive disease transmission. This information will be important evidence for the Indonesian Ministry of Health in planning effective disease control programmes and protecting the health of people living in these areas.

## Introduction

### Background and rationale

Land-use changes are one of the main drivers of infectious disease emergence, disrupting ecosystems and increasing opportunities for pathogen spread between people, wildlife and insect vectors ^1^. Within Southeast Asia, one of the largest planned land-use changes is the construction of the new Indonesian capital city Ibu Kota Nusantara (IKN) on the island of Borneo. While the current Indonesian capital city Jakarta is a highly dense urban area with a population of over ten million people ^‎2^, IKN is currently being developed within a rural, mostly forested area. The construction of IKN is still in the phase of basic infrastructure development and the initial relocation of the core government. According to The Handbook of IKN Relocation ^‎3^, the long-term vision plan until 2045 is to build the area into a sustainable, smart and inclusive capital city that supports national governance and encourages new economic growth. However, the roadmap for supporting public health as well as the policy governing this domain has not yet been a top priority.

The relocation of Indonesia's capital city to IKN in East Kalimantan, an area endemic for malaria and dengue, poses significant public health challenges ^‎4^. The population of IKN is projected to grow rapidly, from 147,430 people in 2025 ^5^ to approximately 1.5–1.6 million by 2035 and reaching an estimated 1.7–1.9 million by 2045. This rapid demographic shift will inevitably be followed by the expansion of clearing the forest area, The Handbook estimates around 256,000 hectares (ha) of high-biodiversity hotspots are vulnerable to these ecosystem changes. Rapid human driven changes to land use, climate, including deforestation and urbanisation, can alter vector habitats and human-vector interactions, potentially increasing infectious disease transmission ^1^. Zoonotic malaria, particularly *Plasmodium knowlesi*, is a concern in areas undergoing extensive deforestation, as seen in nearby Sabah, Malaysia ^‎6– 9^. However, data on the impact of environmental changes on malaria (especially *P. knowlesi*) and dengue in East Kalimantan are scarce. While WHO reported over 1,1 million malaria cases nationally in 2022, local data from the Province Health Office of East Kalimantan, the hotspot near IKN contributed the highest number 544 malaria cases in 2024 ^‎10^. Furthermore, there is limited information on other vector-borne diseases, such as chikungunya, Zika, filariasis, and Japanese encephalitis. Current diagnostic tools and surveillance approaches have limitations in these contexts ^11, 12^.

Serological diagnostics, which detect pathogen-specific antibodies, are valuable for assessing population exposure, especially for rare or difficult-to-diagnose infections ^13^. Multiplex screening platforms allow simultaneous assessment of exposure to numerous pathogens ^14– 16^. Previous research indicates that antibody responses to multiple malaria antigens can effectively stratify transmission levels and predict outbreak-prone areas in elimination settings in Indonesia ^16^. Similarly, recently published studies have shown that specific dengue antigens can identify serotypes of past infection and can be used to study serotype-specific exposures and transmission dynamics ^‎17^.

Leveraging this unique opportunity to monitor the impact of land use changes on infectious disease dynamics within a large-scale development, we aim to assess the distribution of malaria and arbovirus exposures in the surrounding areas and populations living and working within IKN. Critically, this will enable us to establish baseline levels of exposure in resident populations and assess differences with populations within the new development. To achieve this, this study will utilize a novel combination of antigens to assess exposure levels to multiple malaria species ( *P. falciparum*, *P. vivax* and *P. knowlesi*) and dengue virus serotypes ( *DENV-1*, *DENV-2*, *DENV-3* and *DENV-4*). Linking these multi-disease antibody responses with demographic, clinical, and environmental data will enhance understanding of malaria and dengue transmission dynamics in and around IKN.

### Aims and objectives

The overall aim of the study is to investigate transmission dynamics of malaria and dengue in the IKN development area, Penajam Paser Utara District, East Kalimantan Province, Indonesia. The study specific objectives include:

a. To assess seroprevalence, spatial patterns, and demographic and environmental risk factors for infections from multiple malaria species ( *P. falciparum*, *P. vivax*, *P. knowlesi*);
b. To assess seroprevalence, spatial patterns, and demographic and environmental risk factors for infections from dengue and its four serotypes ( *DENV-1*, *DENV-2*, *DENV-3*, and *DENV-4*);
c. To assess RDT-based incidence, spatial patterns, and demographic and environmental risk factors of malaria infections.

## Protocol

### Study area

The study will be conducted at sites that have reported malaria and dengue infections in IKN and the surrounding areas in Penajam Paser Utara District, East Kalimantan Province, Indonesia. IKN has a total land and water area of approximately 324,332 ha. The water area covers 68,188 ha, while the land area covers 256,142 ha, consisting of two areas, namely the Nusantara Capital Region (KIKN) and the Nusantara Capital Development Region (KP-IKN). The development of the IKN has progressed over an area of 56,160 hectares and will continue according to the targeted plan where the selected villages are included in KP-IKN ^‎18^. The landscape of IKN consists mainly of built-up areas, roads, buildings, bare ground, and some tree cover. The villages in surrounding areas are mostly still forested, with some areas covered by oil palm plantations. The total population of the villages in IKN is more than 15,000, with around 5,000 households while villages in surrounding areas have a population of more than 9,000 with around 3,000 households ( Table 1).

**Table 1.** The total population in each village ^19^.

Villages	Population	Households	Neighborhood Units (RT)
Sotek	6,475	2,285	17
Bukit Subur	975	302	10
Riko	2,149	700	6
Bumi Harapan	2,380	789	10
Tengin Baru	4,306	1,430	22
Suko Mulyo	2,214	758	13
Argo Mulyo	3,387	1,169	24
Semoi Dua	3,407	1,129	23

**Figure 1.**
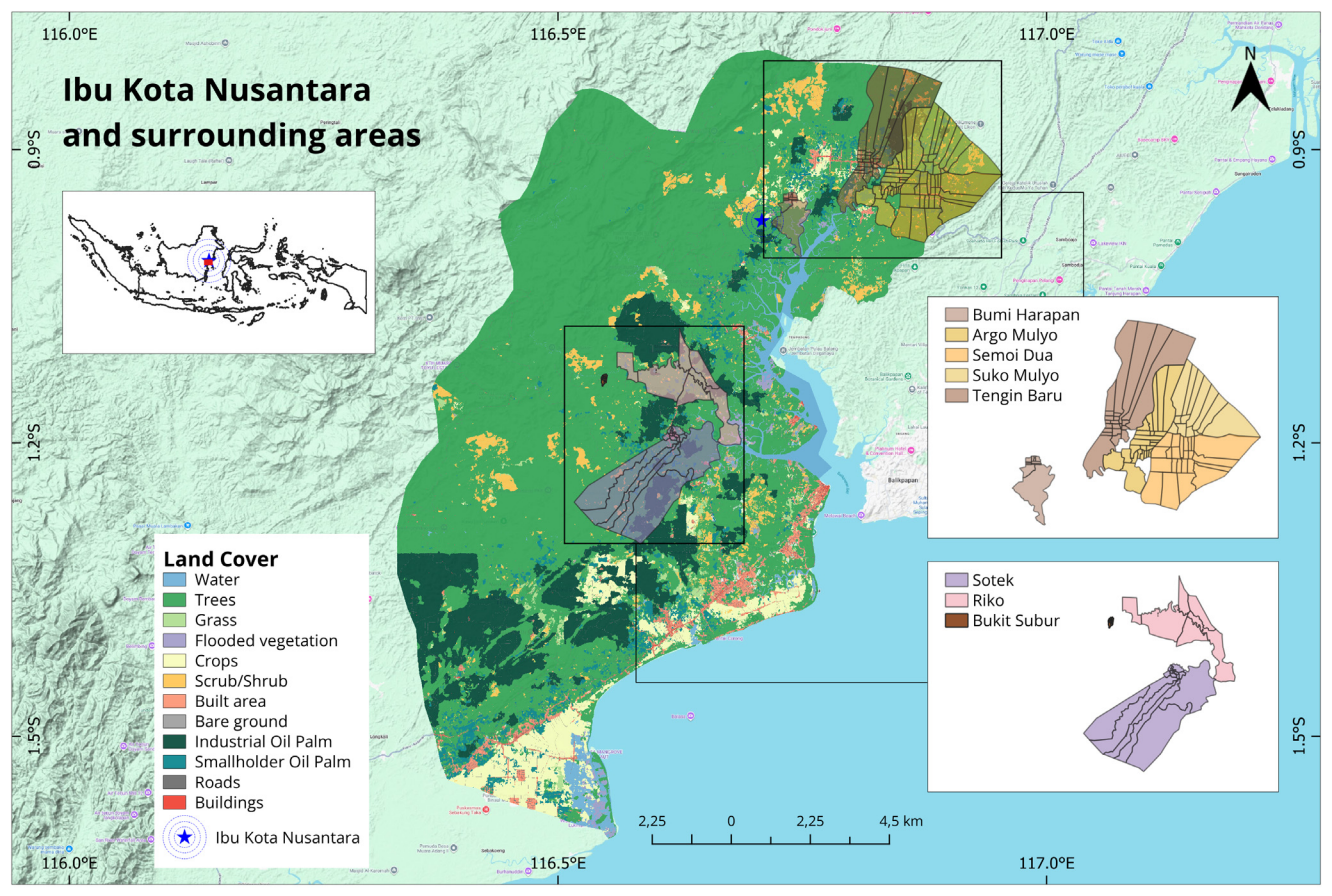
The study location in Ibu Kota Nusantara and surrounding areas, East Kalimantan Province.

Sites will represent areas with a high burden of malaria and dengue, varying levels of deforestation and land-use changes, and population movements associated with the IKN development. Study sites will include five villages in IKN (Bumi Harapan, Tengin Baru, Suko Mulyo, Argo Mulyo, and Semoi Dua) and three villages in surrounding area (Sotek, Bukit Subur, and Riko) ( Figure 1). All selected villages in IKN were classified as malaria and dengue-endemic areas. Whilst all villages in surrounding areas were malaria-endemic areas.

### Environmental characterisation

IKN and its surrounding areas, located in East Kalimantan, Indonesia, has a tropical rainforest climate with high humidity and year-round rainfall. The region features lowland hills, river valleys, and coastal plains, with ecosystems ranging from dense tropical forests to mangroves and peatlands. Its biodiversity is rich, home to endangered species like orangutans and sun bears. However, much of the natural habitat has been degraded due to logging, oil palm, agriculture, coal mining, and expanding infrastructure, particularly in connection with the development of Ibu Kota Nusantara, Indonesia’s new capital city ^20^.

Environmental pressures in the study sites include deforestation, peatland conversion, and habitat fragmentation, which threaten ecological stability and local livelihoods. Rivers and wetlands play a critical role in the region’s hydrology, but are vulnerable to pollution and disruption. As development accelerates, balancing ecological conservation with urban expansion is a key challenge, especially in maintaining biodiversity, managing land use sustainably, and safeguarding the interests of indigenous and local community.

### Ethical considerations and permissions

Ethical Clearances were obtained from the Health Research Ethics Committee, Faculty of Medicine, Lambung Mangkurat University in South Kalimantan (Approving Number 022/KEPK-FKIK ULM/EC/IV/2025) and the Oxford Tropical Research Ethics Committee (Approving Number 1744484). This study will be conducted in full accordance with these ethical guidelines. All participation will be voluntary. Trained field workers will obtain written informed consent from all adult participants and from the parents or legal guardians of participating minors (aged >1 year). Assents will be obtained from minors aged 14–17. Specific consent will also be sought for the long-term storage of specimens. All participant data and samples will be anonymized using a unique coding system to ensure confidentiality. Participants who test positive for malaria via RDT will be referred to the local primary health care facility for free treatment, as per national guidelines.

### Sample size

Approximately 250 randomly selected participants aged ≥1 year old will be enrolled in each site, resulting in a total of 2,000 samples. For the serological survey, a minimum sample size of 248 individuals per site will be sufficient. This calculation is based on the methodology for estimating the malaria antibody seroconversion rate (SCR) from age-specific seroprevalence data ^‎21^, which allows an SCR as low as 0.0036 to be estimated with a precision level of +/- 0.0018.

To ensure representativeness, we will employ a multistage random sampling approach ^‎22^, beginning with stratification at the village/urban (desa/kelurahan) level, with each village acting as an independent stratum. A total of 250 respondents will be targeted per stratum, assuming each household (HH) has approximately three eligible participants. Based on this assumption, approximately 84 households are required per village (250 respondents / approximately 3 participants per HH). For the total study of 2,000 respondents across eight villages, the total number of households needed is approximately 667 (2,000 respondents / 3 participants per HH).

In the first stage (primary cluster sampling), neighborhood units (RT) will be selected using probability proportional to size (PPS) based on the number of households in each RT. The number of RTs selected per village (n) is determined by the average number of households per RT. If the average is ≥50, then 5 RTs are selected; if it is <50, the number of RTs selected is the minimum between the total number of RTs and 10.

The selection of RTs will use a systematic sampling interval method. The probability of selecting RT *i* is calculated as $P_{i}=\frac{M_{i}}{M_{total}},$ where *M _i_* is the number of households in RT- *i*, and *M _total_* is the total number of households in the village. The selection procedure involves: (a) creating a sampling frame of RTs with household counts, (b) calculating cumulative household totals, (c) determining the sampling interval $k=\frac{M_{total}}{n},$ (d) randomly selecting a starting number *r* between 1 and *k*, and (e) selecting RTs whose cumulative totals included *r, r + k, r + 2k, ..., r + (n –1)k*.

In the second stage (secondary cluster sampling), households will be selected from each sampled RT. The allocation of households per RT (denoted as *m*) is calculated as the total number of households per village divided by the number of RTs selected. The floor value *m _floor_* is first assigned equally, and any remaining households are randomly allocated (+1) across randomly selected RTs. If the total number of households in a selected RT is less than the assigned *m*, all households in that RT will be selected, and the shortfall will be adjusted in another RT.

### Household census

Meetings will be held with community leaders and members to introduce study activities and address any questions. Following initial meetings, all households within the survey areas will be enumerated and assigned a unique identification number linked to the household’s GPS coordinates. If no household members are present at the time of the survey, fieldworkers will revisit the household or attempt to contact them by phone to schedule an appointment.

### Serological surveys

Households selected during the sampling and all individuals who resided in the selected household the previous month will be asked to participate in the survey. The study will be explained by a trained research fieldworker and written informed consent will be obtained during the household visit. Consenting individuals will be interviewed on demographic characteristics, movement patterns, travel history, health seeking behavior and land-use practices. Electronic questionnaires will be designed using KoboToolbox ( www.kobotoolbox.org) and data will be collected using encrypted electronic tablets. A combination of village meetings and call-backs will be used to trace non-responders.

**Figure 2.**
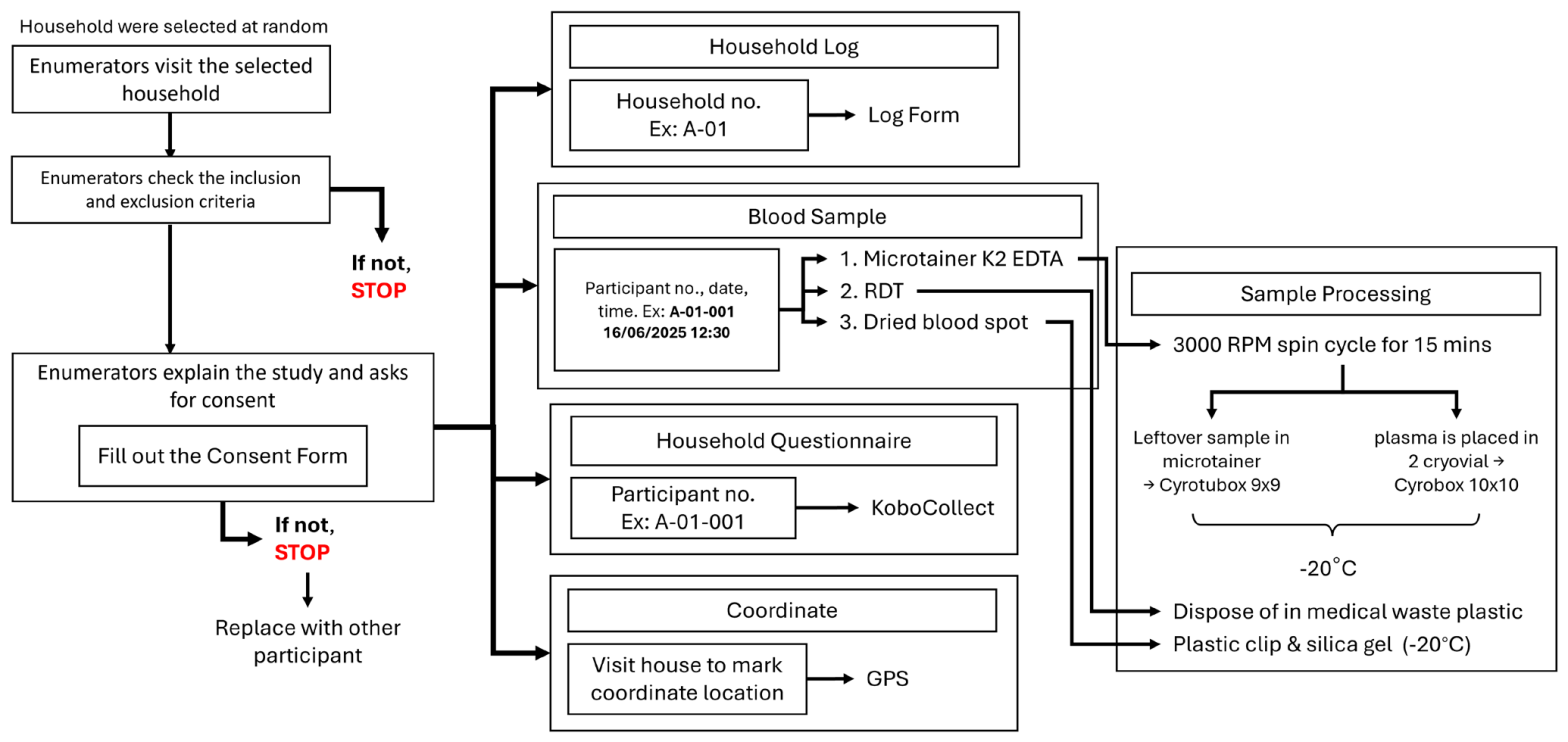
Workflow of study activities.

A trained fieldworker will perform finger-prick blood sampling to collect a maximum of 500μl of whole blood using an EDTA micro-container and three blood spots (~20μl each) on filter paper ( Figure 2). Blood samples will be used for serological analysis of exposure to malaria and dengue. Additionally, we will include other pathogens of interest to the Indonesian Ministry of Health. The whole blood and filter paper bloodspots will be collected and stored at the - 20°C freezer located at each site, until transferred to the - 80°C freezer at OUCRU Indonesia, Jakarta. Malaria rapid diagnostic tests (RDTs) will be performed immediately for all consenting individuals. Participants with positive test results will be referred to local primary health care facilities where they will have free access to treatment and management in accordance with standard Indonesian Ministry of Health guidelines.

Individual written informed consent will be obtained from all participants and/or their legal guardians. An information and consent form in clear, simple language will be provided to the participants. If the participant is younger than the legal majority (18 years) consent will be sought from his/her relatives and/or legal representative. For minors who can give assent (14 to 17 years of age), the study investigator should obtain assent in addition to the legal representative’s consent. The potential subject’s dissent should be respected. The investigator’s responsibility is to conduct the informed consent interview, devote sufficient time to addressing any participant questions, and to obtain consent from each participant or his/her parents and/or legal representative before any sample or any data is collected. Two original ICFs must be completed, dated and signed personally by the participant or his/her parents and/or legal representative and by the investigator. The participant will receive one signed original form; the investigator will retain the second original.

For minors, able to give assent, two original Informed Assent Forms must be completed, dated and signed personally by the participant and the investigator. If the participant (or parent or legal representative) is unable to read, a relative or an impartial witness should be present during the informed consent discussion. The participant should give consent orally and, if capable, complete, sign and personally date the information and consent form. The witness must then complete, sign and date the form together with the investigator. Any changes to the data collection and sampling procedures that affect the participant’s involvement will be communicated to the participant. If a participant decides to withdraw from the study, their data and information will be purged from the database and their samples will also be destroyed.

The amount of blood to be collected by finger-prick is an adequate volume for the tests (malaria RDT and Luminex assay). Adverse health risks associated with blood sampling in this study include the sensation of a pin-prick as blood is drawn from the finger. Participants will immediately get information on their malaria RDT test. If the RDT results are positive, participants will be referred to the nearest primary health care facility for standard malaria treatment. Laboratory results and epidemiological data collected in this study will be shared to local and national control malaria programs to support their surveillance control programs. Ultimately, the information generated by this study will support ongoing efforts to reduce infectious burdens and improve population health in Indonesia.

### Environmental data collection

We will additionally assemble open-source satellite-derived data on key environmental and spatial factors throughout this time period, including land cover ^23^, forest cover ^‎24^, elevation ^25^, population density ^‎26^ and climate variables ^‎27^.

### Laboratory tests

Blood samples will be analysed against pre-validated antigens for *P. falciparum, P. vivax, P. knowlesi* malaria, *DENV-1, DENV-2, DENV-3* and *DENV-4* dengue viruses, and other pathogens of interest to the Ministry of Health, using bead-based assays ^28^. This laboratory work will be conducted at OUCRU Indonesia laboratory in Jakarta. The dengue multiplex assays are developed at Duke-NUS Medical School in Singapore, whilst the malaria assays are developed at the London School of Hygiene and Tropical Medicine in the UK. Components of the assay will be transferred to OUCRU Indonesia laboratory for serology data generation with the study samples.

### Specimen storage

Specimens may be stored for up to ten years with patient consent. These samples will be used for serological analysis and further research may include characterizing infection, exposure to other pathogens and refinement of laboratory methods. Any further research using these samples must be approved by all Institutional Review Boards. If at any point the subject withdraws consent for the storage of specimens, the specimens will be destroyed, and the data will not be used. All participants will be provided with contact details for the study in the patient information sheet if they wish to withdraw consent. Samples will be identified only by anonymized codes and cannot be linked to identifiable information (see Confidentiality). Specimens will be stored in secure freezer facilities at OUCRU Indonesia for up to 10 years.

### Statistical analysis plan

We will describe patterns in species-specific malaria incidence, and antibody responses to malaria and dengue by age, gender, geographical location, and other potential risk factors. Different methods for identifying cut-off for seropositivity to each antigen, such as finite-mixture model and machine learning approaches, will be explored. We will assess the age-specific prevalence of species-specific antimalarial antibodies and antibodies against different dengue virus serotypes. Specifically, to measure the population-level exposure to each malaria species, the force of infection, also referred to as the seroconversion rate (SCR), will be estimated by fitting a reverse catalytic model to age-specific seroprevalence data for each species ^29^. Models allowing two forces of infection will be fitted if deemed a better fit, using likelihood ratio methods.

The antibody responses data will be integrated with spatial and demographic data to examine the relationships between exposure patterns and population characteristics, and to subsequently generate spatial risk surfaces for malaria and dengue exposure. Mixed-effects logistic regression models will be used to examine risk factors associated with seropositivity to each malaria species and dengue virus serotypes. Variables with evidence of an association (p < 0.05) in bivariable analysis will be included in a multivariable model. The site will be treated as a random effect in both bivariable and multivariable models. Spatial analysis will be performed to identify areas with clustering of high antibody responses for each disease. Geostatistical models of household seroprevalence will be fitted in a Bayesian framework, modelling the probabilistic dependence of latent (unobserved) variables using population-level antibody responses, environmental and spatial factors ^‎30^.

### Dissemination

We will disseminate outputs to researchers and key stakeholders at subnational, national, and regional levels. Subject to ethical restrictions, all data, associated metadata, data collection protocols, and statistical code will be uploaded and shared via a GitHub repository and published as supplements to open access journal publications to ensure reproducibility.

To improve vector-borne diseases surveillance and control programs, we will ensure that results and findings from this study are shared with relevant stakeholders, as soon as they are available. The Ministry of Health will be provided with initial data as soon as they are available and will be updated on the results of subsequent analyses. Results will be published as soon as this study is complete. Guidelines for authorship of international peer-reviewed journals will be used to establish authorship. All results will be published in open-access journals and presented to the Ministry of Health and the World Health Organization. We will additionally share research outputs to wider audiences through networks such as the Asia Pacific Malaria Elimination Network.

## Conclusions

This study will be an essential assessment in serological analysis in Ibu Kota Nusantara and surrounding area by directly observing how massive, planned land use changes are associated with vector-borne diseases, particularly malaria and dengue. Through integrating satellite-derived environmental data, this study will provide new insights about the baseline levels of immunity between the community in the area of development and wider population to effectively support health programmes in the Indonesian’s new capital city.

## Data Availability

No data associated with this article. Monash University Bridges. Ibu Kota Nusantara and surrounding areas. https://doi.org/10.26180/30978415 ^31^. The project contains the following extended data: SeroVec Questionnaire EN_version 1.0_6 March 2025.pdf. (Questionnaire form) 001 Informed Consent - EN.pdf. (Informed consent procedure) 002 Interview Guide - EN.pdf. (Interview procedure) 003 GPS Collection - EN.pdf. (Collecting household coordinate procedure) sls_sotek_fixed.shp. (Neighborhood unit in Sotek Village) sls_bukit_subur_fixed.shp. (Neighborhood unit in Bukit Subur Village) sls_riko_fixed.shp. (Neighborhood unit in Riko Village) sls_bumi_harapan_fixed.shp. (Neighborhood unit in Bumi Harapan Village) sls_tengin_baru_fixed.shp. (Neighborhood unit in Tengin Baru Village) sls_suko_mulyo_fixed.shp. (Neighborhood unit in Suko Mulyo Village) sls_argo_mulyo_fixed.shp. (Neighborhood unit in Argo Mulyo Village) sls_semoi_dua_fixed.shp. (Neighborhood unit in Semoi Dua Village) PPU_Land_Cover_2020_CSIRO.tif. (Land cover of IKN and surrounding areas) Data are available under the terms of the Creative Commons Attribution 4.0 International license (CC-BY 4.0).
