# Supplementary material for: A study protocol for a baseline community-based cross-sectional study to investigate malaria and dengue transmission dynamics in a development area of new capital city, Indonesia
Source: Wellcome Open Res. 2026 Feb 3;11:89. [Version 1] doi: 10.12688/wellcomeopenres.25303.1 (PMC12966794; doi:10.12688/wellcomeopenres.25303.1)
Supplement: Peer review history

# Reviewer report: Reviewer response for version 1

**DOI:** 10.21956/wellcomeopenres.27886.r146601

**Published:** 2026-03-06

**Authors:** Harapan Harapan

**Affiliations:** 1 Universitas Syiah Kuala, Banda Aceh, Aceh, Indonesia

**Overall assessment**

 Overall, this is a well-designed study protocol with a robust methodological approach. The multistage sampling strategy and the statistical analysis plan are particular strengths. The issues identified are minor and with these minor revisions, the protocol should be ready for indexing and implementation.

**Abstract**

- In the Abstract, the eligibility criterion is written as “individuals aged >1 year,” whereas other sections use “aged ≥1 year.” Please choose one format (either “>” or “≥”) and apply it consistently throughout the manuscript.
- Please explicitly state the main objective(s) of the study in the Background portion of the Abstract.

**Claims about dengue serotyping and formatting**

- The statement: *“To achieve this, this study will utilize a novel combination of antigens to assess exposure levels to multiple malaria species (P. falciparum, P. vivax and P. knowlesi) and dengue virus serotypes (DENV-1, DENV-2, DENV-3 and DENV-4).” *raises a concern. Please clarify whether the proposed platform can reliably differentiate dengue virus serotypes (DENV-1 to DENV-4). The currently cited studies (14–16, 28) do not appear to support serotype-level discrimination, based on the information provided. Please confirm and revise the claim as needed. Or any available study assessing its performance compared to serotyping PCR. 
  In addition, “DENV-1” to “DENV-4” should not be italicized.
- The sentence: *“Similarly, recently published studies have shown that specific dengue antigens can identify serotypes of past infection and can be used to study serotype-specific exposures and transmission dynamics.17” *Please support this statement with at least two appropriate references.  

**Aims and Objectives**

 In Objective (c), the phrase “RDT-based incidence” appears conceptually inconsistent with a cross-sectional design, which typically estimates prevalence rather than incidence. If incidence is intended, please specify the follow-up structure (e.g., repeated measurements over time) and clearly describe how incidence will be measured. If repeated assessments are not planned, consider revising “incidence” to “prevalence” (or an equivalent cross-sectional measure) for accuracy.

**Table 1**

 The title of Table 1 should be expanded to be more complete and self-explanatory (i.e., understandable without referring extensively to the main text).

**Study area definitions**

 In the sentence, “Sites will represent areas with a high burden of malaria and dengue, varying levels of deforestation…”, please define “high burden” and “varying levels of deforestation” more explicitly in this section. For example, specify the data source and threshold(s) used to categorize burden, and the metric(s) used to quantify deforestation (and how categories are determined).

**Figure 1 (map clarity)**

 Figure 1: The map is somewhat confusing. It is unclear what the dark green color represents—does it indicate tree cover? If so, what does the surrounding lighter area represent (e.g., grassland )? It is uncertain whether such clear boundaries between trees and grass actually exist on the ground as depicted in the map. 

**Subheading wording**

 Consider renaming the subheading “Sample size” to **“Sample size and sampling method”** to better reflect the content and improve readability.

**Redundancy in consent description**

 In the Serological Surveys section, the paragraph describing written informed consent appears to repeat information already provided in the Ethical Considerations section. Please consider condensing the text and/or referring back to the Ethics section to reduce redundancy while maintaining clarity.

Is the study design appropriate for the research question?

Yes

Is the rationale for, and objectives of, the study clearly described?

Yes

Are sufficient details of the methods provided to allow replication by others?

Partly

Are the datasets clearly presented in a useable and accessible format?

Yes

Reviewer Expertise:

Virology and Public Health

I confirm that I have read this submission and believe that I have an appropriate level of expertise to confirm that it is of an acceptable scientific standard, however I have significant reservations, as outlined above.

# Reviewer report: Reviewer response for version 1

**DOI:** 10.21956/wellcomeopenres.27886.r149749

**Published:** 2026-03-06

**Authors:** Peter Asaga Mac

**Affiliations:** 1 Universitatklinikum, Institute of virology, Freiburg, Germany

**Peer Review of the Study Protocol**

**A study protocol for a baseline community-based cross-sectional study to investigate malaria and dengue transmission dynamics in a development area of new capital city, Indonesia.**

**Overall assessment**

 The manuscript outlines a community-based cross-sectional serological study intended to examine malaria and dengue transmission patterns in the rapidly developing Indonesian capital region of Ibu Kota Nusantara (IKN). The topic is timely and of potential policy relevance. Large-scale environmental transformation, particularly deforestation and urban expansion, has long been associated with altered vector ecology and shifting disease dynamics. Establishing baseline serological data in such a setting could therefore prove useful.

 That said, the protocol occasionally reads more as a programme description than as a fully specified epidemiological design. Certain methodological elements appear under-developed, and some conceptual distinctions particularly between exposure, infection, and transmission are not always clearly maintained. The study is plausible in principle, yet several sections would benefit from greater analytical precision and stronger justification of methodological choices.

**Title and abstract**

 The title communicates the nature of the work and identifies the study design clearly. However, the phrase “development area of new capital city” feels somewhat imprecise and stylistically awkward. A more direct formulation referring explicitly to the Ibu Kota Nusantara development region would likely read more naturally.

 The abstract provides a succinct summary of the study’s objectives and general approach. Nevertheless, a conceptual inaccuracy appears in the phrasing “malaria and other arboviruses.” Malaria is caused by a protozoan parasite rather than an arbovirus, and this wording may confuse readers unfamiliar with vector-borne disease classifications. The issue is minor but should be corrected.

 The methodological description is broadly acceptable but somewhat vague. The abstract mentions statistical and geostatistical models without specifying the modelling framework or analytical objectives. While brevity is expected in this section, the wording gives the impression that the analytical plan has not yet been fully defined.

 The plain language summary is generally clear. However, it somewhat overstates what serological evidence can reveal. Antibody detection reflects past exposure rather than current infection or precise transmission timing, and the wording could acknowledge this distinction more cautiously.

**Introduction and rationale**

 The introduction situates the study within the broader literature linking land-use change to infectious disease emergence. This framing is appropriate. The discussion of the Indonesian capital relocation provides useful policy context and highlights the scale of the planned environmental transformation.

 At the same time, certain claims are asserted rather than substantiated. The manuscript suggests that public health considerations have not been a major priority within the capital relocation programme. This may well be accurate, but the statement would benefit from documentary support or policy references.

 The epidemiological context for malaria is presented rather briefly. The manuscript notes that 544 malaria cases were reported near IKN in 2024, yet the reader is not given a clear sense of whether this number represents a substantial burden relative to other regions or historical trends. Without comparative context, the significance of the figure is difficult to interpret.

 Dengue epidemiology receives even less quantitative discussion, despite being a central focus of the study. Including recent dengue incidence data or outbreak history in East Kalimantan would strengthen the rationale considerably.

 The discussion of multiplex serological assays is generally appropriate but occasionally conflates surveillance and diagnosis. Multiplex bead assays detect antibodies and are primarily useful for estimating past exposure. They are not diagnostic tools for active infection, and the terminology should reflect this distinction.

**Aims and objectives**

 The study objectives are clearly presented, although they are somewhat repetitive. The first two objectives mirror each other for malaria and dengue, which is understandable but perhaps unnecessarily duplicative.

 More importantly, the manuscript does not fully articulate how the proposed serological measurements will inform understanding of transmission dynamics. Cross-sectional serosurveys can provide valuable estimates of historical exposure, yet they cannot directly measure incidence or real-time transmission without strong modelling assumptions. A brief acknowledgement of this limitation would improve conceptual clarity.

 The reference to “RDT-based incidence” raises methodological questions. Rapid diagnostic tests detect current infection but cannot measure incidence in a single cross-sectional survey unless repeated measurements are conducted. The wording therefore appears imprecise.

**Study area description**

 The description of the study setting provides useful demographic and geographical information. Table 1 summarises the population size and number of households across the selected villages, which helps clarify the sampling frame.

 The accompanying map showing land cover categories across IKN and surrounding areas is also helpful. The figure illustrates the mixture of forested land, plantations, and built environments in the region and provides a useful spatial reference for readers.

 However, the environmental characterisation occasionally drifts into general ecological description rather than focusing on epidemiologically relevant features. The discussion of biodiversity and endangered species, while interesting, is only loosely connected to the study’s objectives. A more targeted discussion of mosquito ecology, breeding habitats, or hydrological conditions might strengthen the relevance of this section.

**Sampling strategy**

 The proposed multistage random sampling approach using probability proportional to size is methodologically sound in principle. However, several aspects of the sampling description require clarification. The assumption that each household contains approximately three eligible participants appears somewhat arbitrary. The manuscript does not cite census data or previous surveys supporting this estimate. If the assumption proves inaccurate, the achieved sample size could diverge from the target.

 The description of the neighbourhood unit selection process includes mathematical notation that appears partially incomplete in the text. The probability formula for selecting RT-level clusters is difficult to interpret and may have suffered formatting errors during manuscript preparation.

 In addition, the protocol does not discuss potential sampling biases arising from population mobility. Given the construction activity associated with the new capital, temporary or migrant populations may be common and could be poorly represented in a household-based sampling frame.

**Data collection procedures**

 The procedures for household visits, interviews, and blood sampling are described in considerable operational detail. The workflow diagram depicting the sequence of household selection, consent procedures, and sample processing is particularly helpful in clarifying field activities.

 Electronic data collection using KoboToolbox and GPS-linked household identifiers represents good practice in field epidemiology and should facilitate spatial analysis.

 Nonetheless, the manuscript devotes extensive space to administrative aspects of consent documentation while providing relatively limited information about the questionnaire variables themselves. For instance, demographic characteristics, mobility patterns, occupational exposures, housing conditions, and vector control practices could all influence malaria and dengue risk, yet these variables are only briefly mentioned.

 Greater specificity regarding the data collection instruments would improve transparency and reproducibility.

**Laboratory methods**

 The laboratory component relies on multiplex bead-based assays to detect antibodies against multiple malaria species and dengue serotypes. This is a reasonable approach for estimating population-level exposure patterns.

 However, the protocol does not discuss the well-known issue of antibody cross-reactivity among dengue serotypes and other flaviviruses. This phenomenon can complicate interpretation of serological results and should at least be acknowledged as a limitation.

 The manuscript also notes that additional pathogens of interest to the Indonesian Ministry of Health may be included in the assay panel, but these pathogens are not specified. Even provisional information would enhance clarity and allow readers to better understand the intended scope of the laboratory analysis.

**Statistical analysis plan**

 The statistical analysis plan outlines several relevant approaches but remains somewhat general. The manuscript mentions finite mixture models and machine learning techniques for defining seropositivity thresholds, yet it does not specify which algorithms will be applied or how model performance will be evaluated. Similarly, the reverse catalytic model proposed for estimating seroconversion rates is an established method in sero-epidemiology. However, the manuscript does not describe how goodness-of-fit will be assessed, how uncertainty will be quantified, or how competing models will be compared.

 The description of geostatistical modelling is particularly brief. While the protocol states that Bayesian models will be used to generate spatial risk surfaces, important methodological details such as spatial covariance structures or prior assumptions—are not discussed.

 Protocols typically include such details precisely so that other researchers can replicate the analytical approach.

**Ethical considerations**

 The ethical framework of the study appears appropriate and well documented. Ethical approvals from Indonesian and international institutional review boards are clearly reported.

 The consent procedures for adults and minors are described thoroughly, and the protocol includes provisions for illiterate participants and specimen storage consent. The plan to refer individuals with positive malaria RDT results for treatment according to national guidelines is also appropriate. One aspect that could be clarified concerns governance of stored specimens. While the protocol states that samples may be retained for up to ten years, the procedures for approving secondary analyses are described only briefly.

**Dissemination**

 The dissemination strategy emphasises open access publication and sharing of data and code through public repositories. This commitment to transparency is commendable and aligns with contemporary expectations in global health research. However, the dissemination plan remains somewhat general. It would be helpful to specify the anticipated format of released datasets or the timeline for public data availability.

**Conclusion**

 The manuscript describes a potentially valuable baseline serological survey in a rapidly transforming region where environmental change may influence vector-borne disease dynamics. The study could contribute useful information for surveillance planning in Indonesia’s new capital. Nevertheless, several aspects of the protocol require clarification before the study design can be fully evaluated. The epidemiological context could be strengthened, the sampling assumptions require clearer justification, and the statistical analysis plan would benefit from greater methodological specificity. Addressing these points would improve both the scientific rigour and the practical reproducibility of the protocol.

**Overall recommendation**: **Minor revision**

 The study question is well chosen and the fieldwork plan is broadly feasible, but the protocol is not yet as specific as it should be for a methods-facing publication. If the authors tighten terminology (exposure vs infection vs incidence), add basic comparative epidemiological context for both malaria and dengue in the study area, specify the “other pathogens” likely to be included, and expand the statistical analysis plan with concrete modelling choices and assumptions, the manuscript should meet a high protocol standard. If those elements remain vague, it risks being judged as only partly reproducible and therefore less useful to other researchers.

Is the study design appropriate for the research question?

Yes

Is the rationale for, and objectives of, the study clearly described?

Yes

Are sufficient details of the methods provided to allow replication by others?

Partly

Are the datasets clearly presented in a useable and accessible format?

Yes

Reviewer Expertise:

Molecular and clinical virology and emerging infectious diseases. molecular biology and immunology and infectious diseases, vaccine research and immunopathology, clinical research management and international health, public health epidemiology and disease surveillance.

I confirm that I have read this submission and believe that I have an appropriate level of expertise to confirm that it is of an acceptable scientific standard, however I have significant reservations, as outlined above.

# Reviewer report: Reviewer response for version 1

**DOI:** 10.21956/wellcomeopenres.27886.r146605

**Published:** 2026-02-24

**Authors:** Adam T Craig

**Affiliations:** 1 The University of Queensland, Brisbane, Australia

**Thank you for the opportunity to review your manuscript. Please find comments and suggestions below.**

**Minor Comment 1**

 In the abstract, the manuscript refers to “malaria and other arboviruses.” As malaria is not caused by an arbovirus, this wording may unintentionally confuse readers. A minor edit in the abstract — and similarly in paragraph two of the introduction where the same phrasing appears — should readily address this.

**Minor Comment 2**

 The second paragraph provides a helpful overview of the malaria and dengue situation in and around IKN. To further strengthen this section, the authors might consider (a) including a dengue-related statistic alongside the malaria case numbers, and (b) where possible, providing context for the malaria figure reported. For example, is 544 cases considered high relative to other provinces or nationally? Such comparison would help readers better interpret the local burden.

**Minor Comment 3**

 In paragraph three, MBA testing is described in a way that may benefit from clarification. MBA measures antibodies and is therefore one form of serological testing; other serological approaches measure antigen. A brief clarification would avoid potential misinterpretation. In addition, it may be worth considering whether “diagnostic” is the most appropriate descriptor for MBA in this context. For several pathogens, MBA remains primarily a research and surveillance tool rather than a routine diagnostic modality.

**Minor Comment 4**

 Regarding the aims and objectives, it would be helpful to clarify whether there are plans to cross-validate malaria MBA findings with results from RDTs or other established diagnostic approaches. If such validation is planned, explicitly stating this as part of the study aims may strengthen the protocol.

**Minor Comment 5**

 The manuscript notes that “additional pathogens of interest to the Indonesian Ministry of Health” will be included in the panel. It would be valuable to specify which pathogens are anticipated, even if provisional, to enhance clarity and reproducibility.

**Minor Comment 6**

 The eligibility criterion stating “all individuals who resided in the selected household the previous month” may benefit from clarification. Does this refer to individuals for whom the household is their usual place of residence, or those who spent at least one night there during the reference period? Clarifying this will improve methodological precision.

**Minor Comment 7**

 The methods are generally well described. The authors may wish to consider whether including specific details of equipment (e.g., brand of filter paper, type of micro-containers) would add practical value for readers intending to replicate or adapt the approach in similar settings.

**Notable Comment 1**

 I defer to the authors’ expertise in arboviral serology; however, it may strengthen the manuscript to acknowledge (a) the evolving and, in some contexts, experimental nature of dengue antibody analysis for surveillance purposes, and (b) the well-recognised challenges of cross-reactivity across dengue serotypes and other flaviviruses. Briefly outlining these considerations would demonstrate awareness of interpretive limitations.

**Notable Comment 2**

 The manuscript would benefit from greater detail regarding the demographic and environmental variables to be collected. Even indicative examples would enhance transparency. The authors might also consider including draft data collection tools or forms as supplementary material, which would increase the utility of the paper for others designing similar studies.

**Notable Comment 3**

 The statistical analysis plan could be strengthened by specifying the analytical methods in greater detail, including the statistical tests to be used and key parameters or assumptions. Providing this level of detail would improve clarity and reproducibility. The authors may find it helpful to consult existing published protocol papers as examples of the level of detail typically included in this section.

Is the study design appropriate for the research question?

Yes

Is the rationale for, and objectives of, the study clearly described?

Yes

Are sufficient details of the methods provided to allow replication by others?

Partly

Are the datasets clearly presented in a useable and accessible format?

Not applicable

Reviewer Expertise:

Surveillance system design; Serosurveillance

I confirm that I have read this submission and believe that I have an appropriate level of expertise to confirm that it is of an acceptable scientific standard.
